# Automatic multiple zebrafish tracking based on improved HOG features

**DOI:** 10.1038/s41598-018-29185-0

**Published:** 2018-07-18

**Authors:** Yun-Xiang Bai, Shu-Hui Zhang, Zhi Fan, Xing-Yu Liu, Xin Zhao, Xi-Zeng Feng, Ming-Zhu Sun

**Affiliations:** 1 Institute of Robotics and Automatic Information System, Tianjin Key Laboratory of Intelligent Robotics, Nankai University, Tianjin, 300350 China; 2 State Key Laboratory of Medicinal Chemical Biology, College of Life Science, Nankai University, Tianjin, 300071 China

## Abstract

As an excellent model organism, zebrafish have been widely applied in many fields. The accurate identification and tracking of individuals are crucial for zebrafish shoaling behaviour analysis. However, multi-zebrafish tracking still faces many challenges. It is difficult to keep identified for a long time due to fish overlapping caused by the crossings. Here we proposed an improved Histogram of Oriented Gradient (HOG) algorithm to calculate the stable back texture feature map of zebrafish, then tracked multi-zebrafish in a fully automated fashion with low sample size, high tracking accuracy and wide applicability. The performance of the tracking algorithm was evaluated in 11 videos with different numbers and different sizes of zebrafish. In the Right-tailed hypothesis test of Wilcoxon, our method performed better than idTracker, with significant higher tracking accuracy. Throughout the video of 16 zebrafish, the training sample of each fish had only 200–500 image samples, one-fifth of the idTracker’s sample size. Furthermore, we applied the tracking algorithm to analyse the depression and hypoactivity behaviour of zebrafish shoaling. We achieved correct identification of depressed zebrafish among the fish shoal based on the accurate tracking results that could not be identified by a human.

## Introduction

Zebrafish have been widely applied in many fields as an excellent model organism, for example in biological experiments^1^, drug screens^2^, gene screens^3^, neurotoxicology analysis^4^, and swarm intelligence development^5^. The notable shoaling behaviour of zebrafish has garnered widespread interest among scientists. It is crucial to achieve accurate and rapid identification and tracking of individuals for relevant mechanistic analyses of and predictions for ecosystems.

However, multi-zebrafish tracking still faces many challenges, such as similar shape, movement and frequent occlusion^6^. A series of computer vision tracking methods were proposed to solve these problems. These methods generally fall into two categories: the tracking methods based on detection-data association and methods based on identification.

In the first category, researchers extracted the objects from videos first and then associated the positions of the objects continuously to achieve multi-object tracking. In 2008, Nevatia proposed a multi-level data association tracking framework^7^. Dicle *et al*. tracked multi-objects with similar appearances by evaluating the rank similarity of the matrix, which was formed by the trajectory data^6^. This system worked well in numerous fields. Similar methods also include an extended kalman filter^8^. However, this types of tracking methods is more suitable for tracking objects with immutable movement. Thus this method cannot be effectively adapted to zebrafish with uncertain mobility.

In the second category, the main idea is to correctly identify individuals. Then multi-object tracking is performed based on the identification. Researchers have manually labelled the targets, such as with colour labels^9^ and visible implantable elastomer (VIE) tags^10^, to achieve identification in the early stage. However, the process of manual marking is very complicated, and the marks even may affect the social behaviour of the objects. In 2014, Alfonso Pérez-Escudero *et al*. proposed idTracker, which identifies individuals based on fingerprinting features on the back texture of animals^11^. This fully automated tracking method has achieved good tracking performances in many fields. However, the method has high requirements regarding the quality and quantity of samples, given that the fingerprinting feature is not stable. Experimental videos must be sufficiently long to ensure each zebrafish has approximately 2500 image samples, which results in a slow processing speed that cannot be widely used in practice. Therefore, it is necessary to design new image features to realize a multi-zebrafish tracking method.

The Histogram of Oriented Gradient (HOG) algorithm is an image descriptor proposed by Dalal *et al*. to detect human^12^. The HOG algorithm calculates the histogram of gradient directions or edge orientations in local regions and is stable when tracking humans. However, the algorithm is orientation-sensitive to the input images and cannot be applied to zebrafish tracking directly. To improve the orientation-sensitive nature of the algorithm and increase stability, we proposed an improved HOG algorithm to calculate the stable back texture feature map of the zebrafish and track multi-zebrafish in a fully automated fashion.

In this paper, we classified the HOG feature blocks based on the correlation of the zebrafish’s back texture and then outputted the relevant back texture feature blocks spirally from the centre. In addition, the training samples were automatically accumulated based on the trajectory analysis and the improved HOG + SVM (Support Vector Machine) classification mechanism. Finally we established the data association strategies based on the identification of multiple zebrafish and obtained the final whole trajectories.

To verify the validity of the improved HOG features, we compared the proposed improved HOG algorithm with the previous HOG method by identifying multiple zebrafish. Then we applied the tracking algorithm to identify 3 types of animal models, and all of them were successfully identified. A plurality of zebrafish in the growth cycle more than a month was also identified. Furthermore, eleven videos of different numbers and different sizes of zebrafish were processed. The tracking results showed that the accuracy rates were considerably increased compared with state-of-the-art tracking methods. Statistics of 8 tracking videos revealed that the average accuracy rate of the proposed method is 99.27%, 4.33% higher than idTracker^11^. In the Right-tailed hypothesis test of Wilcoxon, our method performed better than idTracker, with significant higher tracking accuracy. Throughout the video of 16 zebrafish, the training sample of each fish had only 200–500 image samples, one-fifth of the idTracker’s sample size. The proposed method can also calculate the stable feature of zebrafish larvae, and have a good tracking performance. Finally, we applied the tracking algorithm to analyse the depression and hypoactivity behaviour of the zebrafish shoaling. We achieved correct identification of depressed zebrafish among the fish shoal based on accurate tracking results that could not be identified by a human. In summary, the proposed method based on improved HOG features tracks multiple zebrafish in a fully automated fashion with low sample size, high tracking accuracy and wide applicability.

## Methods and Materials

**Figure 1:**
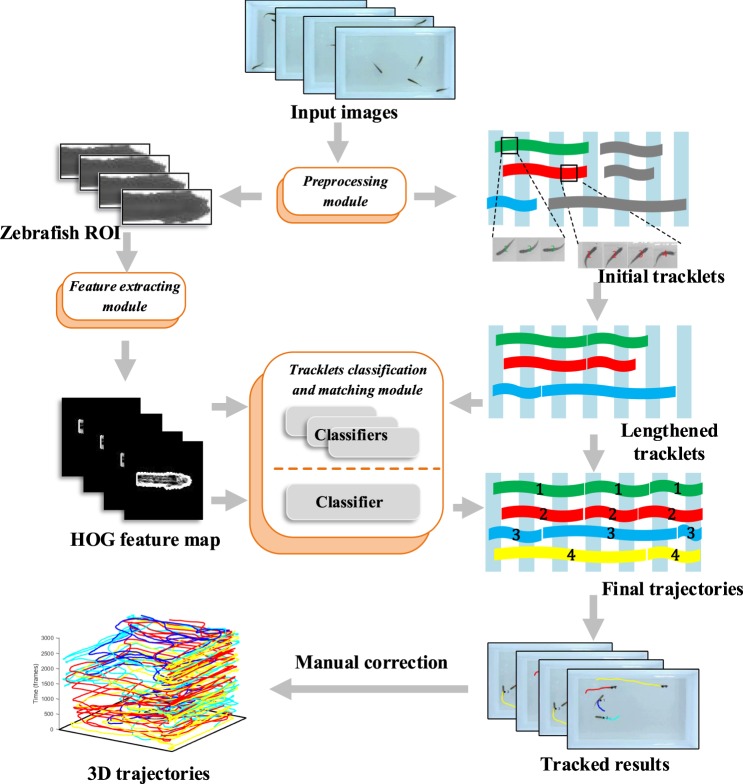
Overview of the proposed multiple zebrafish tracking system. There are three main modules: the Preprocessing module, the Feature extracting module, the Tracklets classification and matching module. In the Preprocessing module, we gathered ROIs of single zebrafish as image samples for classifier and matched the zebrafish positions into initial tracklets. In the Feature extracting module, we proposed the improved HOG algorithm, then we extracted the improved HOG features of zebrafish image samples. In the Tracklets classification and matching module, we trained 2 types of classifiers by applying the improved HOG+SVM mechanism. The initial tracklets before and after the same crossings were further lengthened based on the first type of classifier to enrich the image samples. The second type of classifier was trained by the longest tracklets of each zebrafish among the lengthened tracklets to obtain the final trajectory.

The tracking algorithm consists of three modules: the Preprocessing module, the Feature extracting module, and the Tracklets classification and matching module (Fig. 1). In the Preprocessing module, we gathered ROIs of single zebrafish as image samples for classifier and matched the zebrafish positions into initial tracklets, which are short trajectory segments for each individuals with timing relations. In the Feature extracting module, we proposed the improved HOG algorithm and then extracted the improved HOG features of zebrafish image samples. In the Tracklets classification and matching module, we trained 2 types of classifiers by applying the improved HOG + SVM mechanism. For the first type of classifier, we trained numerous classifiers by the initial tracklets before and after the same crossings. Then, we lengthened the initial tracklets by this type of classifiers to enrich the image samples. The second type of classifier was trained by the longest tracklets of each zebrafish among the lengthened tracklets. We used the only classifier to obtain the final trajectory of each zebrafish.

### Preprocessing module

The Preprocessing module contains two parts: zebrafish ROI image samples collection and initial tracklets acquisition. On one hand, we built the background model and intercepted the zebrafish ROIs as image samples. On the other hand, we matched the zebrafish positions into initial tracklets for each individual as long as possible.

### Zebrafish ROI collection

**Figure 2:**
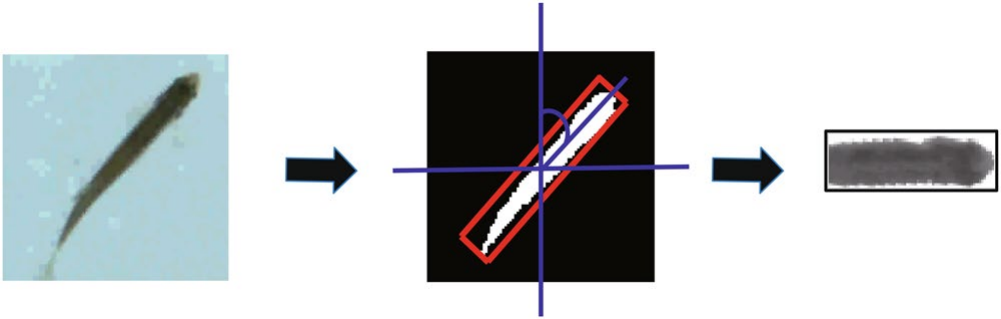
The flow chart of zebrafish ROIs collection. We intercepted the head region as the zebrafish ROI by calculating the minimum enclosing rectangles, the center coordinate and the tilt angle *θ* of the whole zebrafish region.

The flow chart of zebrafish ROI collection is presented in Fig. 2. We used a static background model for motion detection, given that the laboratory environment is relatively stable. The average intensity values of the first *f* frames were used to calculate the intensity of the background model:

1 $$Bm(x,\,y)=\sum _{t=1}^{f}{G}_{t}(x,y)/f$$

where *G*_*t*_*(x*,*y*) is the intensity at point (*x*,*y*) on frame *t*. We extracted the whole zebrafish region by applying the background subtraction method. Then we obtained the minimum enclosing rectangles of the zebrafish in the whole zebrafish region by using an elliptic fitting algorithm, and calculated the centre coordinate and the tilt angle *θ* of the enclosing rectangle. The initial value of *θ* is ranged from −90° to 90°.

We intercepted the head region of the zebrafish as the zebrafish ROI given that the whole zebrafish is a non-rigid object and the shape may change as it swims. To obtain the stable HOG feature, we set a threshold *Thr*_*e*_ of the aspect ratio of the enclosing rectangle to exclude the zebrafish ROIs where the fish bodies are significantly deformed or overlapping occurs, shown as Figs 1 and 2 in Supplement s1. Finally, we rotated the zebrafish ROI to orient the fish head to the right, which greatly eliminated the orientation-sensitity of the HOG algorithm.

**Figure 3:**
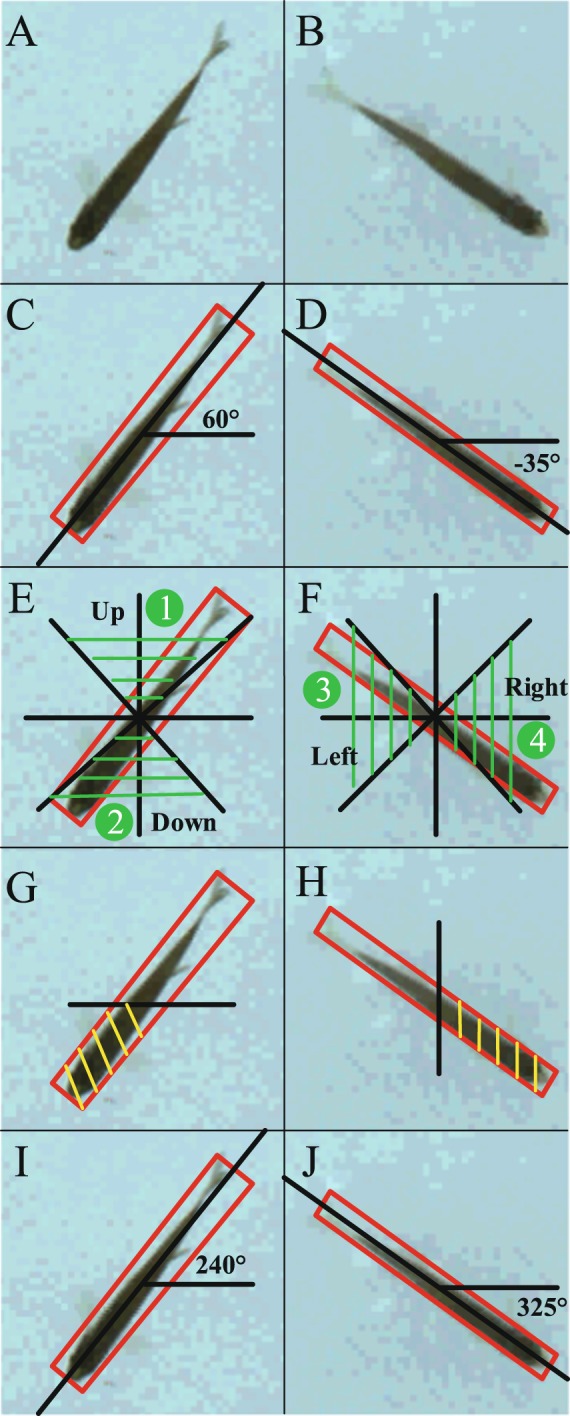
Angle calculation of zebrafish head. (**A**,**B**) The original image. (**C**,**D**) Calculate the parameters of the whole zebrafish region based on the elliptic fitting. (**E**,**F**) Determine the direction of zebrafish body. The zebrafish is oriented towards left and right if *θ* ∈ (−45°, 45°), as the region ③ and ④ in F, and the zebrafish is oriented towards up and down otherwise, as the region ① and ② in E. (**G**,**H**) Count the sum of pixels and get the head position. The head existed on the side of the larger sum pixels of the external rectangle region. (**I**,**J**) Recalculate the tilt angle of the zebrafish in the range of [0, 360°] based on the head direction.

As shown in Fig. 3, the steps of zebrafish ROI extraction are listed as follows:

1. Determine the direction of the zebrafish body. The zebrafish was oriented towards the left and right if *θ* ∈ (−45°, 45°) (regions ③ and ④ in Fig. 3F). Otherwise, the zebrafish was oriented up and down (regions ① and ② in Fig. 3E).
2. Calculate the position of the zebrafish head, *pHead*. The head is positioned on the side with the larger sum of pixels in the external rectangle region, as shown in Fig. 3G,H.
3. Recalculate the tilt angle of the zebrafish in the range of [0, 360°] based on the head direction as shown in Fig. 3I,J.
  2 $$\theta =\{\begin{array}{ll}\theta  & if\,\theta  > {\rm{0}}^\circ \,\& \,pHead\,\in \,region\,\mathrm{\textcircled{1} }\,or\,\mathrm{\textcircled{4} }\\ \theta +180^\circ  & if\,\theta  > {\rm{0}}^\circ \,\& \,pHead\,\in \,region\,\mathrm{\textcircled{2} }\,or\,\mathrm{\textcircled{3} }\\ \theta +360^\circ  & if\,\theta  < {\rm{0}}^\circ \,\& \,pHead\,\in \,region\,\mathrm{\textcircled{2} }\,or\,\mathrm{\textcircled{4} }\\ \theta +180^\circ  & if\,\theta  < {\rm{0}}^\circ \,\& \,pHead\in region\,\mathrm{\textcircled{1} }\,or\,\mathrm{\textcircled{4} }\end{array}$$
4. Rotate the enclosed rectangle region clockwise to orient the fish head to the right based on the modified tilt angle *θ*.
5. Intercept the head to aggregate the zebrafish ROIs collection.

### Initial tracklets acquisition

We applied a heuristic strategy based on the minimum distance to obtain the initial tracklets without object overlapping because the swimming displacement of fish is limited in consecutive frames. Thus, the length of initial tracklets is related to the crossing frequency in approximately 50–300 frames.

Let *O*_*i*,*t-1*_ and *O*_*j*,*t*_ denote a zebrafish on frame *t-1* and frame *t*, respectively, and *N*_*t-1*_ and *N*_*t*_ are the numbers of objects on frame *t-1* and frame *t*, respectively. *D(O*_*i*,*t-1*_, *O*_*j*,*t*_*)* denotes the Euclidean distance between *O*_*i*,*t-1*_ and *O*_*j*,*t*_. Then the matching results are:

3 $$m({O}_{i,t-1},\,{O}_{j,t})=\{\begin{array}{ll}1 & if\,j={\rm{\arg }}\,\mathop{{\rm{\min }}}\limits_{j}(D({O}_{i,t-1},\,{O}_{j,t}))\\ 0 & otherwise\end{array}$$

If two objects *O*_*i*,*t-1*_ and *O*_*k*,*t-1*_ on frame *t-1* matched with the same object *O*_*j*,*t*_ on frame *t* according to equation (3), we set:

4 $$\{\begin{array}{c}m({O}_{i,t-1},\,{O}_{j,t})=0\\ m({O}_{k,t-1},\,{O}_{j,t})=0\end{array}$$

Additionally, the numbers of objects would change if the zebrafish were crossing, so the crossing determination criteria is defined as follows:

5 $$C({O}_{i,t-1},\,{O}_{j,t}))=\{\begin{array}{ll}0, & if\,{N}_{t-1}={N}_{t}\\ 1, & otherwise\end{array}$$

In particular, *C*(*O*_*i*,*t-1*_, *O*_*j*,*t*_) = 0 while both *O*_*i*,*t-1*_ and *O*_*k*,*t-1*_ matched with *O*_*j*,*t*_, which may be caused by the stationary fish or the special matching distance. Then we matched *O*_*i*,*t-1*_ and *O*_*j*,*t*_ if the difference between *D(O*_*i*,*t-1*_, *O*_*j*,*t*_) and *D(O*_*k*,*t-1*_, *O*_*j*,*t*_) is greater than threshold *Thr*_*d*_:

6 $$if\,D({O}_{k,t-1},\,{O}_{j,t})-D({O}_{i,t-1},\,{O}_{j,t}) > Th{r}_{d}\Rightarrow \{\begin{array}{c}m({O}_{i,t-1},\,{O}_{j,t})=1\\ m({O}_{k,t-1},\,{O}_{j,t})=0\end{array}$$

We discarded the short tracklets (<10 frames) caused by crossings to avoid introducing the possible overlapping errors.

### Feature extracting module

In the Feature extracting module, we proposed the improved HOG algorithm to calculate the stable back texture feature map of zebrafish, which greatly increased the certainty of identification. Then we extracted the improved HOG features of zebrafish image samples.

### Improved HOG feature extracting

The basic idea of the HOG algorithm is that local object appearance and shape can often be characterized rather well by the distribution of local intensity gradients or edge directions^12^. It is implemented by dividing the binary image into small spatial regions (“cells”), for each cell accumulating a local 1-D histogram of gradient directions or edge orientations over the pixels of the cell. There is no overlapping between the cells. For better invariance to illumination, shadowing, etc., a certain number of cells are configured to form the larger spatial regions (“blocks”). Then a measure of local histogram “energy” is generated over blocks and the results are used to normalize all of the cells in the block. Overlapping between the blocks is noted. Finally, the feature vectors are output by sliding windows. However the algorithm has orientation-sensitity to the input images.

**Figure 4:**
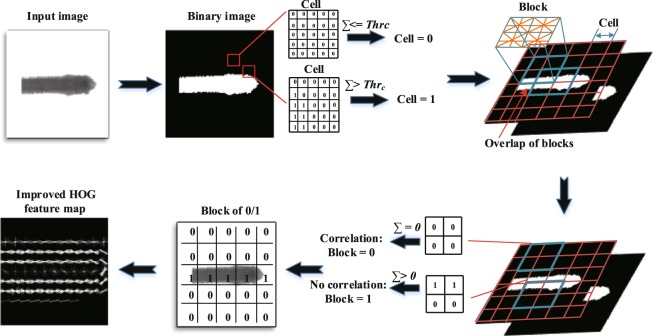
The process of the improved HOG algorithm. First we applied the HOG algorithm to convert the zebrafish image samples to the HOG feature map. Then we classified the HOG feature blocks based on the correlation of the zebrafish’s back texture. The fixed number of blocks marked as 1 were outputted spirally from the centre. Finally we obtained the improved HOG feature with low dimensionality but high stability.

**Figure 5:**
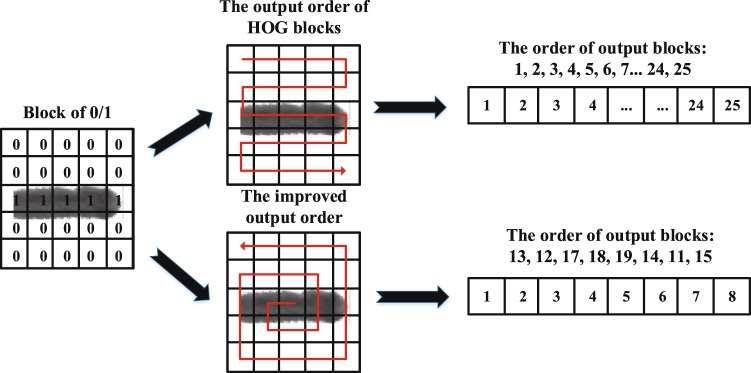
The order of output blocks in the HOG feature map. We spirally output the fixed number of blocks marked as 1 to calculate the final HOG feature, which contains more texture features and less background. The size of the feature is small compared with the previous method.

To improve the orientation-sensitive nature of the algorithm and reduce the feature dimension, we proposed the improved HOG algorithm. We scaled the zebrafish ROIs to a specified size as the image sample, such that the input images of the improved HOG algorithm with same size are consistent in resolution, which improves the adaptability of image resolution. Then we classified the HOG feature blocks based on the correlation of the zebrafish’s back texture. Block 0 indicates there is no correlation between the block and the back texture, block 1 indicates they are relevant. Then, a fixed number of blocks marked as 1 was outputted spirally from the centre to obtain the improved HOG feature with low dimension but high stability. Figure 4 presents the process of the improved HOG algorithm. The steps are as follows:

1. Scale the zebrafish ROIs to the specified size *P*_*r*_ as the image sample.
2. Binarize the image samples. The foreground pixels and the background pixels are marked as 1 and 0 respectively. Let *Bi*_*t*_*(x*,*y)* be the binary value of point *(x*,*y)* on frame *t*:
  7 $$B{i}_{t}(x,\,y)=\{\begin{array}{ll}1, & if\,{G}_{t}(x,\,y) > average\,(Bm)\\ 0, & otherwise\end{array}$$
  where *Bm* is the background model in equation (1).
3. Apply the HOG algorithm to convert the zebrafish image samples to the HOG feature map.
4. Calculate the pixel number with the value of 1 in each cell of the binary image. The cell is marked as 0 if the pixel number is less than a fixed threshold *Thr*_*c*_.
5. Group all the labelled cells into blocks. The block is marked as 0 if the sum of cells in the block is 0. Otherwise, the block is marked as 1.
6. Output the fixed number of blocks marked as 1 spirally to calculate the final HOG feature as shown in Fig. 5. The fixed number of blocks is set to *P*_*b*_.

### Tracklets classification and matching module

In this module, we trained 2 types of classifiers by applying the improved HOG + SVM mechanism. The initial tracklets before and after the same crossings were further lengthened based on the first type of classifier. We aimed at stitching the tracklets as long as possible here to enrich and accumulate the image samples. A plurality of classifiers was trained in this manner. The other type of classifier was trained to calculate the final trajectory of each zebrafish. It trained by the longest tracklets belonging to each zebrafish without overlapping among the lengthened tracklets.

**Figure 6:**
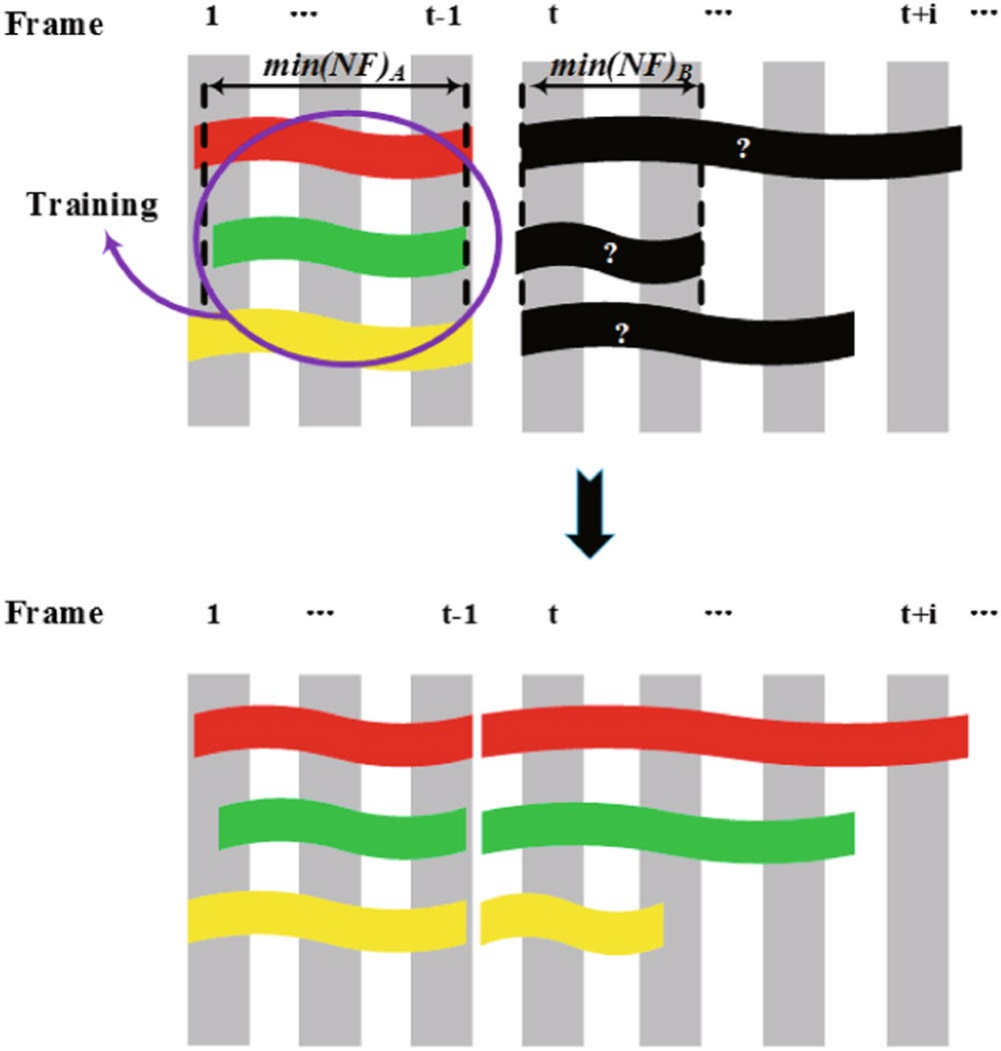
Initial tracklets selection to train the classifier. Assuming that the tracklets disconnected on frame *t*, each colourful tracklet belongs to one identity, and the black tracklets denote the tracklets with unclear identities. The tracklet group before the crossing is denoted as A and the group after the crossing is denoted as B. There are 3 identities in each group. Group A is selected as the training set, and group B serves as the test set as *min(NF)*_*A*_> *min(NF)*_*B*_.

**Figure 7:**
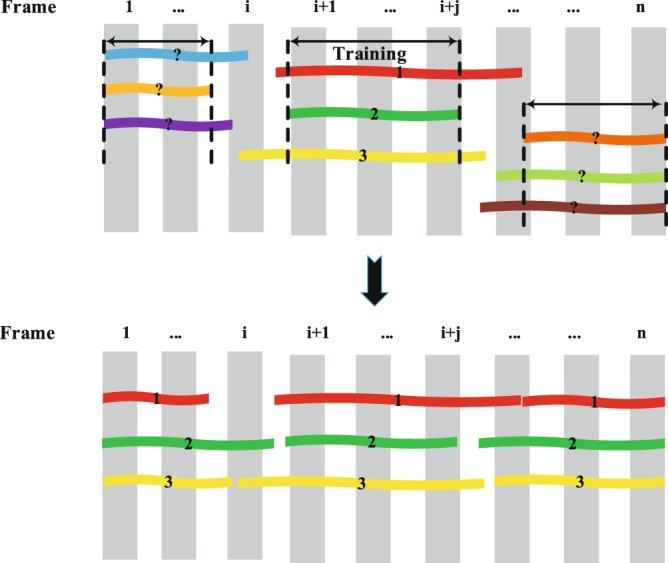
Tracklets classification and matching. When N=3, there are 9 tracklets with 3 identities waiting to be matched. The numbers on tracklets donate the labels. Class the tracklets without the label based on the classifier and match the tracklets with the same label.

Let N be the number of zebrafish tracked in the tracking video. T_t_ = {T^*i*^_*t*_ | i = 1, 2, 3…N} denotes a group of tracklets sharing the same frame t. NF_t_ = {NF^*i*^_*t*_ | i = 1, 2, 3…n} denotes the length of tracklets in group T_t_. In the training of the first type of classifiers, we first calculated the shortest tracklets with length of min(NF_t_) in the two groups of tracklets before and after crossing. Then we compared the lengths of the shortest tracklets of two groups to obtain the longer tracklets. Third, we used group T_t_ with the longer tracklet to train the SVM classifier. Finally, we classified the tracklets in the other group by the classifier and associated the tracklets with the same identities. As shown in Fig. 6, group A is selected as the training set, and group B is employed as the test set as min(NF)_A_ > min(NF)_B_. Then, we stitched the initial tracklets with the same identity. In this manner, the samples were accumulated by the lengthened tracklets, which greatly increases the certain of identification. For training the second type of classifier, many tracklets groups are waiting to be matched, as shown in Fig. 7. We chose one group as the training set for the SVM classifier. First, we calculated the shortest tracklets with a length of min(NF_t_) in all groups. Then we compared these shortest tracklets to obtain the longest tracklets with a length of max(min(NF_t_)). Finally the tracklet group T_t_, which contained the longest tracklet, was selected to train the classifier.

Let *p*_*ij*_ be the predicted probability of *T*^*i*^_*t*_ classified as the class *T*^*i*^_*t+n*_. The probabilities matrix is noted as follows:8Select *N p*_*ij*_ in equation (8), no two of which lie in the same row or column. We used the Hungarian algorithm^13,14^ to match the tracklets group *T*_*t*_ and *T*_*t+n*_ based on *p*_*ij*_. If the sum of *N p*_*ij*_ was maximal and all of the *p*_*ij*_ were greater than *1/N*, matching was performed. The predicted label of the tracklet *T*^*i*^_*t*_ (*i =1*, *2*, *3…N*) are calculated as follows:

9 $$\begin{array}{c}\,\,\,\,\,{L}_{i}=j\\ st.\,{p}_{ij} > \frac{1}{N};\,1 < i < N;\,1 < j < N\end{array}$$

which indicated the matching association is successful if the tracklets before and after the interval have the same label.

We also provided the manual error correction function to correct tracking errors and improve the accuracy of tracking results. To reduce manual checking, the possible tracking errors are located in the image sequence automatically. The error correction is accomplished by convenient human-computer interaction. We assume that the matching error may occur in tracklets when the matching probability value is less than a threshold *Thr*_*p*_. The error correction function automatically locate these points. Then the user can view the tracking results of adjacent frames and correct the wrong matching results easily by clicking the mouse. This function is detailed in Supplement s1.

### Parameters settings

The cell and block sizes in the HOG algorithm were set to 9*9 pixels and 2*2 cells respectively for HOG feature map calculation. In addition, there are 5 threshold values and 3 parameters need to be set in the tracking system, as shown in Table 1. In the first module, *Thr*_*s*_ and *Thr*_*g*_ are the area threshold and grey threshold, respectively, when applying the background subtraction method to extract the entire zebrafish region. *Thr*_*s*_ is determined by the size of the fish body, and *Thr*_*g*_ is set based on the grey scale difference between the foreground and background. *Thr*_*e*_ is the enclosing rectangle aspect ratio threshold of the zebrafish ROI, which is used to to remove the samples with serious body deformation or overlapping. *P*_*r*_ is the scale parameter when scaling the zebrafish ROI to the specified scale as the final image sample. Both values are set based on zebrafish size. *Thr*_*d*_ is the distance threshold of equation (6) in the case of matching redundancy caused by stationary fish or special matching distance. In the second module, *P*_*c*_ is defined as the pixel number in a cell, which determines whether the cell is related to the zebrafish’s back texture, and *P*_*b*_ is the number of spirally outputted blocks in the improved HOG algorithm. In the third module, *Thr*_*p*_ is the probability threshold to determine whether matching errors occur. We tracked 11 videos with different number of zebrafish, different video frame rates and different sizes of zebrafish based on the parameters in Table 1. All of the parameters work well with high validity. In fact, only *Thr*_*s*_ and *Thr*_*g*_ needed to be adjusted in different tracking videos.

**Table 1:** Parameters settings in the experiments.

First module				Second module		Third module	
*Thr*_*s*_	*Thr*_*g*_	*P*_*r*_	*Thr*_*e*_	*Thr*_*d*_	*P*_*c*_	*P*_*b*_	*Thr*_*p*_
500–800	20–50	100*100	3	30	10	34	60%

### Evaluation metrics settings

To evaluate the performances of the multi-Object identification, we defined the Identifying Accuracy (IA), which denotes the probability that the target is correctly identified, and the Classification Accuracy (CA), which denotes the probability that the correct labels predicted by the algorithm. In addition, to quantify the occurrence of crossings in the tracking dataset, we defined that the crossing occurs in the current frame if the detected fish number is less than the total fish number. The CrossFrequency denotes the frequency of crossings. Finally, we set the evaluation metrics calculated as follows to evaluate the performances of the tracking system:

$$IA=\frac{number\,of\,correct\,identified\,fish}{number\,of\,identified\,fish}$$

$$CA=\frac{\sum number\,of\,correct\,predicted\,labels\,of\,each\,fish}{{\sum numberofclassifiedframesofeachfish}^{}}$$

$$CrossFrequency=\frac{total\,frames\,where\,the\,detected\,fish\,number\,is\,less\,than\,the\,total\,fish\,number}{number\,of\,tracked\,frames}AccuracyRate=1-\frac{\sum number\,of\,wrong\,tracked\,frames\,of\,each\,fish\,}{number\,of\,tracked\,frames}$$

$$MissRate=\frac{{\sum }^{}number\,of\,missed\,frames\,of\,each\,fish\,}{number\,of\,fish\,\ast \,number\,of\,frames}$$

$$ErrorRate=\frac{{\sum }^{}number\,of\,wrong\,tracked\,frames\,of\,each\,fish}{number\,of\,fish\,\,\ast \,number\,of\,frames}$$

### Ethics statement

All of the experimental protocols and procedures involving zebrafish were approved by the Committee for Animal Experimentation of the College of Life Science at Nankai University (no. 2008) and were performed in accordance with the NIH Guide for the Care and Use of Laboratory Animals (no. 8023, revised in 1996).

### Data availability

The datasets generated during and analyzed in this research are available on GitHub: https://github.com/deitybyx/hog_ZebraTracker.

## Results

The automatic zebrafish tracking system was implemented in MATLAB (version: 2015b). The codes were run on a platform with a quad-core Intel Core i5-6500, 3.20 GHz CPU, 8GB RAM. We utilized LIBSVM^15^ for SVM classifier. See Fig. 3 in Supplement s1 for more detail of the system interface.

### Experiments

The experiment was performed in an in-house developed observation system^16^. In the observation system, an industrial camera (Microvision, EM-120C) with a resolution of 1280 * 960 was used to capture the videos of zebrafish from the top view. A 25 cm * 15 cm * 15 cm tank was used for 10 or less zebrafish, and a tank of 60 cm * 45 cm * 15 cm was used for greater than 10 zebrafish, with a water depth of 10 cm; the vertical distances between the camera and the bottom of the tank were 50 cm and 100 cm respectively, which ensures the images captured by the camera are clear and legible.

In the experiments, we identified 9 zebrafish to compare the improved HOG algorithm and the previous HOG method. In addtion, we used 3 types of animal models and 11 videos with different numbers of zebrafish to evaluate the performances of the tracking algorithm. Furthermore, we applied the tracking system to analyse the depression and hypoactivity behaviour in zebrafish shoaling.

#### Method comparison

**Figure 8:**
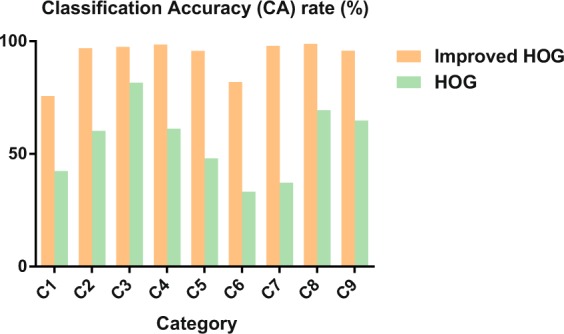
The comparison of 9 zebrafish classification accuracy (CA) rate between the improved HOG algorithm and the previous HOG method. The classification accuracy (CA) rate of each fish computed by the proposed improved HOG algorithm is much higher than the previous HOG method.

We applied the proposed improved HOG algorithm and the previous HOG method to process the same 9 zebrafish. Then, we compared the Classification Accuracy (CA) rate of each zebrafish to evaluate the performances of the two methods. The 9 zebrafish were imaged separately. Each fish video contained approximately 1500 frames. The first 1000 frames in each video were gathered to train the classifier and the last 500 frames of each video were used to test. Category represents the ground truth label of the targets. As shown in Fig. 8, the Classification Accuracy (CA) rate of each fish computed by the proposed improved HOG algorithm is considerably increased. The average Classification Accuracy (CA) rates of the two methods are 93.19% and 55.33%, demonstrating that the proposed method in this paper offers improved performances compared with the previous algorithms.

#### Multi-object identifying

In the multi-objects identification experiment, we captured the single model animal sample images, separately. The number of the zebrafish (AB strain) was 30. Each fish video contained approximately 1500 frames. The first 1000 frames in each video were gathered to train the classifier and the last 500 frames of each video were used to test. The experimental results are presented in Table 2.

**Table 2:** Evaluation of zebrafish identifying.

Category	CA	Category	CA	Category	CA
C1	66.8%	C11	93.5%	C21	90.98%
C2	95.23%	C12	64.02%	C22	92.11%
C3	96.78	C13	88.09%	C23	94.69%
C4	97.74%	C14	89.71%	C24	87.68%
C5	95.33%	C15	75.83%	C25	70%
C6	80.21%	C16	88.11%	C26	81.48%
C7	92.73%	C17	67.47%	C27	92.71%
C8	95.18%	C18	79.6%	C28	93.44%
C9	92.94%	C19	81.82%	C29	89.9%
C10	85.89%	C20	87.95%	C30	91.37%

Based on the results, the identifying accuracy (IA) rate was 100%, and the average classification accuracy (CA) rate was 86.7%. In addition, we also identified two other animal models: drosophila (ISO4) and black mouse (C57 B6). Both of these animals were correctly identified. The results are detailed in Supplement s1Tables 1-2.

**Figure 9:**
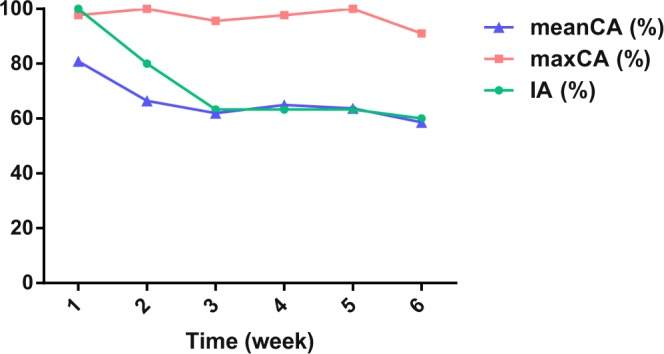
The evaluation of zebrafish identification over the time of the growth cycle. The maximum Classification Accuracy (maxCA) rate was not affected by the growth cycle, demonstrating the stability of the improved HOG features. Further, the Identifying Accuracy (IA) rate and the average Classification Accuracy (meanCA) rate decreased continuously, and the growth tended to be stable after three weeks.

To further verify the stability of the improved HOG feature, we identified 30 zebrafish (10 months old) in the growth cycle for greater than one month. In the experiment, the same 30 zebrafish were tested every week and imaged separately. The sample images of 30 zebrafish collected in the first week were used as a training set for the classifier. Then the classifier was applied to identify zebrafish in future growth cycles. The results are presented in Fig. 9, the Identifying Accuracy (IA) rate and the average Classification Accuracy (meanCA) rate decreased continuously, both of them tended to be stable after 3 weeks. Further, eighteen fish were still identifiable with the average Classification Accuracy (CA) rate of 60% in the 6th week. The maximum Classification Accuracy (maxCA) rate was not affected by the growth cycle. Our algorithm can maintain a relatively high classification accuracy rate over a longer observation period, which proves the stability of the improved HOG features. See Supplement s1 Table 3 for more detailed data.

#### Multi-object tracking

**Figure 10:**
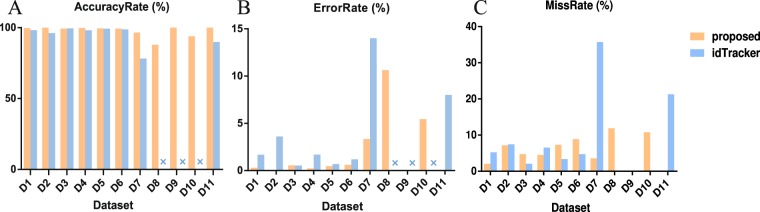
Evaluation of tracking results for different methods. Under the same experimental dataset, the proposed method generally exhibits an increased tracking AccuracyRate and reduced ErrorRate compared with idTracker. The MissRate value obtained by idTracker was smaller in datasets D3, D5 and D6, as we ignored the short tracklets (<10 frames) produced by long time overlapping to avoid extra error. idTracker could not handle the datasets of D8, D9 and D10 due to the short video length, large fish numbers and poor video quality, which are denoted as (×). The proposed method can also calculated the stable feature of zebrafish larvae, with good tracking results in D11, better than idTracker.

In the multi-object tracking experiment, we compared the proposed method and idTracker by tracking 11 videos with different numbers of zebrafish. D9 is the experimental video in^11^, D10 was chose from^17^, D11 was the zebrafish larvae image sequence of dataset 01 in^14^. Dataset information is presented in Table 3. The frame rate of D11 is (*), given that D11 is the zebrafish larvae image sequence. The tracking results are presented in Fig. 10, and more detailed information regarding the results is presented in Supplement s1 Table 4. IdTracker could not utilize the datasets of D8, D9 and D10 due to the short video length, large fish numbers and poor video quality, shown as (×) in Fig. 10. In the same experimental dataset, the proposed method generally exhibits increased tracking AccuracyRate values and reduced ErrorRate values compared with idTracker. Statistics of D1-D7 and D11 demonstrate that the average AccuracyRate of the proposed method is 99.27%, 4.33% higher than idTracker. We performed a paired Wilcoxon test on the values of AccuracyRate from idTracker and our method to show the difference between the overall distributions. In the Right-tailed hypothesis test, the *p-value* of the test is 0.0078 <0.05, the result of the hypothesis test *h* is 1, which indicated that the median of AccuracyRate in our method is significant greater than the median of AccuracyRate in idTracker. The proposed method can also calculated the stable features of zebrafish larvae, with good tracking results in D11, better than idTracker.

**Table 3:** Information of datasets.

Dataset	FishNum	TotalFrame	FrameRate/fps	FishSize/pixel	CrossFrequency
D1	4	3240	13	500–1000	13.67%
D2	5	3596	13	500–1000	26.08%
D3	6	1884	20	500–1000	28.50%
D4	7	1896	13	500–1000	34.49%
D5	8	1647	13	500–1000	28.84%
D6	9	1107	13	500–1000	46.52%
D7	16	1791	15	200–600	29.70%
D8	25	1791	15	200–600	65.55%
D9	5	968	32	250–500	7.23%
D10	25	1081	30	800–1500	48.55%
D11	4	280	*	100–200	8.2%

In the proposed method, the training sample of each fish in D7 included only 200–500 image samples, but the identification still worked well, one-fifth of the idTracker’s sample size, which demonstrates the stability of the improved HOG features. Although the video quality of D9 and D10 were very poor due to video compression, good tracking results were obtained. As noted from the results of the proposed method, when the number of zebrafish and the probability of crossing significantly increases, the AccuracyRate exhibited a decreasing trend (Fig. 10A), and ErrorRate values increased (Fig. 10B). Thus, the AccuracyRate and ErrorRate were severely affected by the frequency of crossings. The MissRate, which is affected by the swimming behaviours of the fish, did not exhibit a certain regularity as the number of fish increased (Fig. 10C), given that the captured images in some videos were unclear as “ghosting”, the constant frame rate cannot meet the changes in the movement states for restless, high mobility and high swimming speed of zebrafish, which affected the stability of the improved HOG feature.

The same experimental datasets were processed by idTracker and the tracking results are also presented in Fig. 10. As the number of objects increased significantly, the AccuracyRate of idTracker was significantly reduced (Fig. 10A). In addition, the MissRate values obtained by idTracker were reduced in datasets of D3, D5 and D6 (Fig. 10C), given that we ignored the short tracklets (<10 frames) produced by long time overlapping to avoid extra error.

#### Shoaling behaviour of zebrafish

We conducted a behavioural analysis of zebrafish shoaling behaviour using the tracking system. Reserpine depletes monoamines, and causes depression and hypoactivity in humans and rodents^18^. We observed whether the depression and hypoactivity in single zebrafish introduced by reserpine manifested in the fish group.

A reserpine (purity ≥ 98.0%) concentration of 40 μg mL^−1^ was chosen in this study based on previous research concerning effective doses of reserpine on depressive behaviour in zebrafish. A total of 108 adult zebrafish (12 months old, male:female = 1:1) were divided into 2 groups: “Reserpine” and “WT”. Nine replicates were conducted for each group. Five wild type zebrafish and 1 target zebrafish were allocated to the “Reserpine” group, and 6 wild type zebrafish were allocated to the “WT” group. In the “Reserpine” group, nine target zebrafish were exposed in the reserpine solution for 20 minutes, and treated zebrafish were maintained in system water for 7 days to induce depression and anxiety-like behaviour. After 7 days in system water, the locomotion behaviour parameters of the 9 treated fish and the 9 random fish in the “WT” group were measured in a novel tank by the total distance travelled, average velocity, turn angle and angular velocity to assess whether the reserpine works^19^. The results are presented in Supplement s1 Fig. 4. The results demonstrate that the zebrafish model of depression and anxiety-like behaviour in the “Reserpine” group were effective. Then we captured the shoaling behaviour of 2 groups, and 9 replicates conducted were conducted for each group. The captured behavioural trial lasted 5 minutes. In this study, the difficulty is how to distinguish the treated zebrafish from the other wild types. Manual marking is not feasible and may harm the zebrafish, so we identified the treated zebrafish in the shoal based on the tracking system.

To identify the treated zebrafish among the normal zebrafish in the “Reserpine” group, two types of videos were captured: video with and without the treated zebrafish. We first applied the tracking system to the 2 types of videos to obtain the final trajectories. Then, we gathered the improved HOG feature maps from the trajectories of the shoal with the treated zebrafish to train the classifier, and the feature maps obtained from the trajectories of the video without the treated zebrafish served the test sets. Finally we calculate the unmatched label as the label of the treated zebrafish based on the classifier. The results for one replicate are presented in Table 4, which provides the predictive probabilities of the zebrafish in the video without the treated zebrafish (Fish*) classified as the class of the zebrafish in the video with the treated target (C*). In addition, data noted in bold denote the maximum classification probability. Thus the label C2 belongs to the treated zebrafish. In the 9 replicates for the “Reserpine” group, all of the treated zebrafish were correctly identified.

**Table 4:** Predictive probabilities of dosing target identifying.

ID	C1	C2	C3	C4	C5	C6
Fish1	4.13%	0.43%	1.74%	**93**.**26%**	0.22%	0.22%
Fish2	**87**.**50%**	0.48%	0.96%	9.62%	0.96%	0.48%
Fish3	1.85%	3.47%	**85**.**65%**	3.70%	0.69%	4.63%
Fish4	6.01%	4.24%	11.31%	0.35%	**74**.**56%**	3.53%
Fish5	1.43%	0.36%	1.79%	1.43%	1.43%	**93**.**57%**

**Figure 11:**
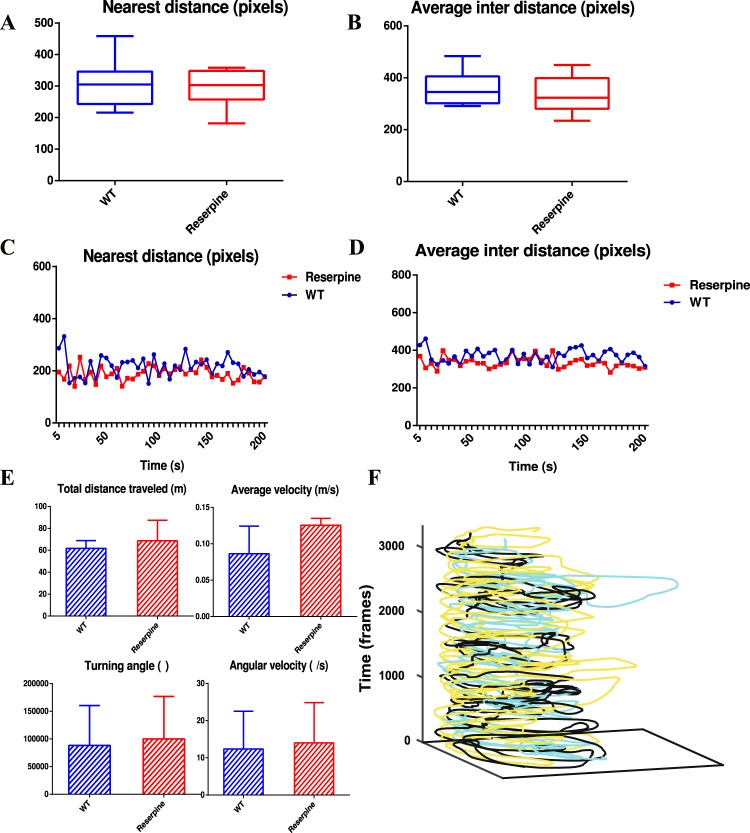
Comparison behaviour parameters between the “WT” group and the “Reserpine” group. (**A**,**B**) The nearest neighbor distance and the average inter-individual distance between the WT group and the Reserpine group are similar. (**C**,**D**) The trends of average and minimum of the inter-individual distance are similar between the WT group and the Reserpine group. (**E**) The locomotion behavior values are similar between the WT group and the Reserpine group. (**F**) In the 3D movement regions of 3 zebrafish in the “Reserpine” group, the black lines denotes the treated zebrafish, whereas the other two lines with different colours belong to different wild type identities. No obvious difference are noted in the movement regions between the treated zebrafish and the normal zebrafish. The treated zebrafish exhibit depression and hypoactivity as single individuals, whereas they perform normally in the fish shoal.

To analyse the behavioural difference between the treated zebrafish and the “WT” group, we calculated the zebrafish shoaling behaviour parameters from the obtained trajectories, including the nearest neighbor distance and average inter-individual distance^20^. The sample images were selected for every 5 s of the complete duration of the recorded trials sampling rate. We calculated the nearest neighbor distance and the average inter-individual distance of 9 replicates in 2 groups, and the average values are presented in (Fig. 11A,B). The nearest neighbor distance and the average inter-individual distance between the “Reserpine” group and the “WT” group are similar in the zebrafish shoal. The trends of nearest neighbor distance and the average inter-individual distance are also similar (Fig. 11C,D).

Additionally, to eliminate fish shoal factors, we calculated the locomotion behaviour parameters of 9 treated zebrafish and 9 random wild types in the “Reserpine” group according to^19^, and all of the values are generally similar between the 2 groups compared with the result in Supplement s1 Fig. 4 (Fig. 11E). Specifically, no obvious difference are noted in the movement regions between the treated zebrafish and the wild types in the “Reserpine” group (Fig. 11F). Although the treated fish perform identically to the wild types, the treated zebrafish exhibited depression and hypoactivity as a single individual, it performed normally in the fish shoal.

## Discussion

To achieve a good performance in tracking, we developed a tracking system to track multiple zebrafish in a fully automated fashion based on an improved HOG feature. The proposed improved HOG algorithm calculated the stable zebrafish’s back texture feature map and increased the certainty of identification. In this paper, we compared the proposed improved HOG algorithm with previous HOG method by identifying multiple zebrafish. Then we successfully achieved the correct identification of 3 types of animal models. In addition, we tracked 11 videos with different numbers and different sizes of zebrafish. In the Right-tailed hypothesis test, the *p-value* of the test is 0.0078 <0.05, the result of the hypothesis test *h* is 1, which indicated that the median of AccuracyRate in our method is significant greater than the median of AccuracyRate in idTracker. In the video of 16 zebrafish, the training sample of each fish contained only 200–500 image samples, only one-fifth of the idTracker’s sample size. These data further demonstrated the stability of HOG features, laying the foundation for the robustness of the tracking system. The proposed method can also calculate the stable feature of zebrafish larvae, and also have a good tracking performance. Furthermore, we applied the tracking system to analyse the depression and hypoactivity behaviour of zebrafish shoaling. We achieved the correct identification of depressed zebrafish among the fish shoal based on the accurate tracking results while humans cannot achieve this level of identification. Based on these behavioural analysis, we inferred that the zebrafish shoal could ease the depression and hypoactivity of a single zebrafish, which can provide a priori information for researchers who want to perform verification experiments. Thus, the proposed method based on an improved HOG feature tracked multiple zebrafish in a fully automated fashion with a low sample size, high tracking accuracy and wide applicability.

The problem of objects overlapping caused by crossings is difficult to address in multi-zebrafish tracking. The method in^17^ utilized the highly accurate object recognition capability of a convolutional neural network (CNN) to distinguish fish of the same congener when crossing occured. Qian Z-M *et al*. proposed a multiple fish 3D tracking method that analysed objects’ motion from three directions simultaneously^21^. Both of these methods provided good tracking performances in the context of crossings. In the future, we will deal the videos from multiple dimensions based on improved HOG features to overcome overlappings. In addition, the method of deep learning is also useful for multi-object identifying, since it automatically calculates the features without manual interaction. We will apply the deep learning combined with the motion parameters to analyse overlapping objects, aiming at improving the tracking accuracy further.

## Electronic supplementary material

Supplement S1 Video

S1 Video

S2

Dataset 1
