# Supplementary material for: Automatic multiple zebrafish tracking based on improved HOG features
Source: Sci Rep. 2018 Jul 18;8:10884. doi: 10.1038/s41598-018-29185-0 (PMC6052047; doi:10.1038/s41598-018-29185-0)
Supplement: Supplementary file 1 — Supplement S1 Video [file 41598_2018_29185_MOESM1_ESM.doc]

# Automatic multiple zebrafish tracking based on improved HOG features

Yun-Xiang Bai^1●^, Shu-Hui Zhang^2●^,Zhi Fan^1^, Xing-Yu Liu^2^, Xin Zhao^1^, Xi-Zeng Feng^2*^, Ming-Zhu Sun ^1*^

^1^Institute of Robotics and Automatic Information System, Nankai University, Tianjin 300350, China.

^2^State Key Laboratory of Medicinal Chemical Biology, College of Life Science, Nankai University, Tianjin 300071, China.

^●^ These authors contributed equally to this work.

* [sunmz@nankai.edu.cn](mailto:sunmz@nankai.edu.cn) (MZS); [xzfeng@nankai.edu.cn](mailto:xzfeng@nankai.edu.cn) (XZF).

## *Supplementary Text S1*

***Supporting information***

**Dataset S1** The tracking system developed in this research written in MATLAB applying to MATLAB015b and above. The file Trace.m is the entry to the GUI interface.

(ZIP)

**Text S1** Methodological details.

(DOC)

**Video S1** Tutorial of 6 zebrafish tracking result.

(WMV)

**Video S2** Tracked result of 16 zebrafish.

(AVI)

***Zebrafish patch collection***

Experiments show that when the fish body has a large bending during swimming, it will affect the global correctness of the back texture of the fish and cause the difference of the further output HOG feature and ultimately affect the correct classification result (Figure 1 and Figure 2). The histogram of oriented gradient is affected by the gray distribution of the fish texture. The gray distribution of the fish texture is different and unstable when fish body deformed. Thus the HOG feature of deformed zebrafish body is disorganized, which is unstable and cannot be identified. Therefore, we calculate the target external rectangle aspect ratio threshold, excluding the case where the fish body was significantly deformed.

**
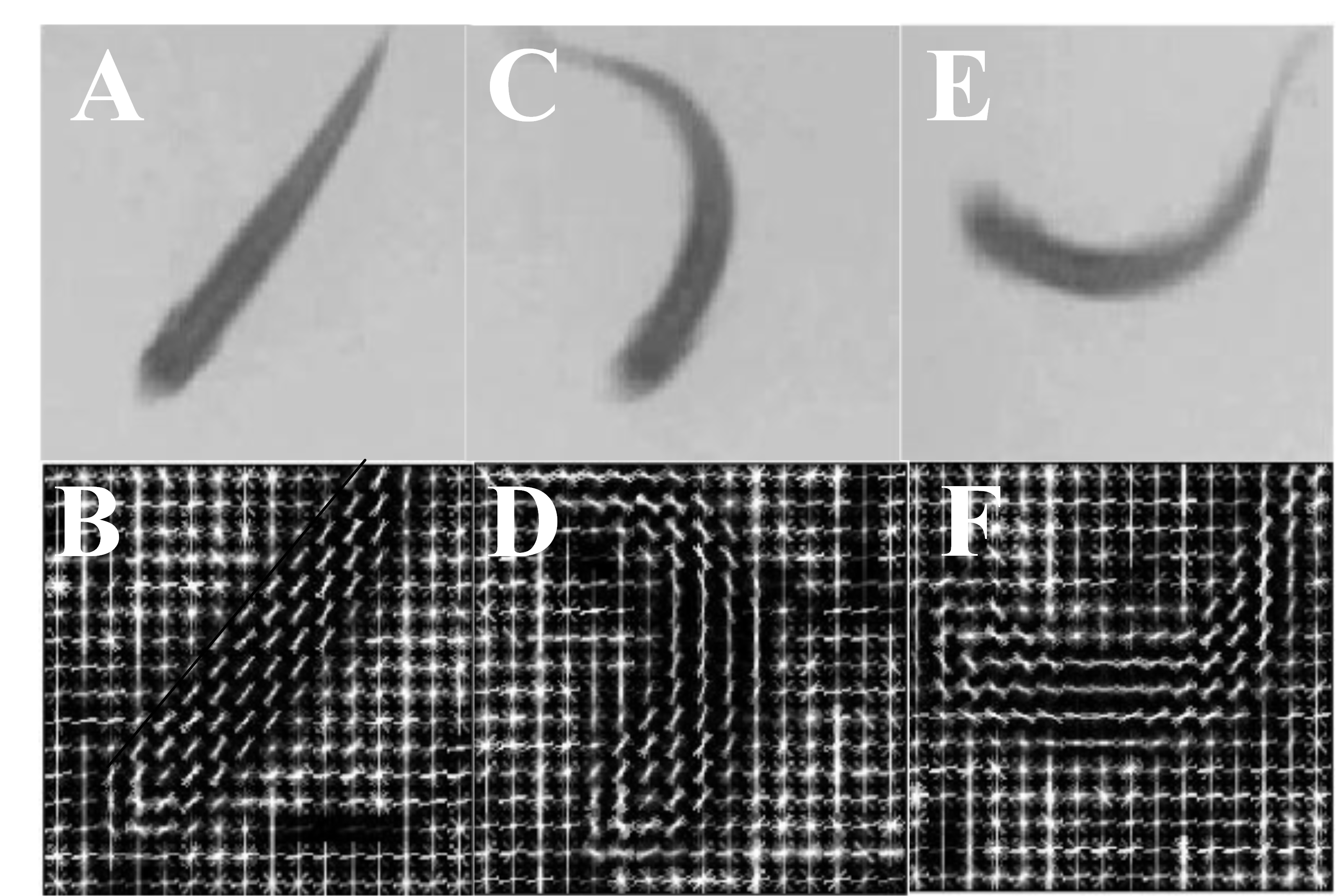
**

**Figure 1. The contrast HOG feature of normal and deformed zebrafish. (A)(C)(E) The grayscale images of different postures of zebrafish. (B)(D)(F) The corresponding HOG feature maps. (A-B) The HOG feature of normal zebrafish body is concise and regular. (B-F) The HOG feature of deformed zebrafish body is disorganized, which is unstable and cannot be identified.**

**Figure 2. The contrast HOG feature of normal and the example with aspect ratio larger than the threshold. (A)(C) The zebrafish ROI of different aspect ratio of zebrafish head region. (B)(D) The corresponding HOG feature maps. (A-B) The HOG feature of normal zebrafish head is regular. (C-D) The HOG feature of example with aspect ratio larger than the threshold is disorganized, which is unstable and cannot be identified.**

***Tracking system***

The tracking system, which is based on the improved Histogram of Oriented Gradient (HOG) feature, provides the easy-to-use graphical user interface and the manual error correction function to increase the correctness of the final trajectories. The GUI interface is shown as Figure **3**. The display interface is on the left, displaying the target center coordinates over the last 20 frames, as well as the initial image sequence; the function panel on the right side is divided into three parts: the Detection Zones, the Tracking Zones and the Results viewing Zones. The detail functions are as follows:

1、The input video is selected by the Load videos button.

2、After calculating the background model, the area threshold and the gray threshold were set for the background subtraction.

3、Previous and Next buttons enable single frame adjustment and positioning of the video.

4、Click the Binary image button to view the background subtraction result, then adjust the value of threshold to determine the optimal parameters.

5、Click the Save parameters button to save the number of targets and the parameters of threshold.

6、There are off-line and on-line modeling mode in the Tracking Zones, and in this paper we applied the on-line mode, needless to pre-shooting the single zebrafish in advance.

7、Click the Tracking button to obtain the final trajectory.

8、The Display trace button and Hide trace button can convert to each other.

9、Click the Pause button to suspend the left result display.

10、The Checkbox can select the fish to add the markup of trace and display on the left interface.

11、Manual correction • OFF button and Manual correction button, as well as the Link trace button can be click-converted by the mouse.

12、The tracklets belonging to different identity with lower confidence are located.

13、Adjust the orders of frames by the Previous and the Next buttons to confirm whether the error occurs. Then click the Accept button to automatically locate the next possible error point.

14、OK button to confirm the manual correction, Cancel button to cancel the current operation.

15、The Message pane displays the message of command windows to remind the user of the current process.

16、The display interface displays the multi-targets trajectory over the last 20 frames.


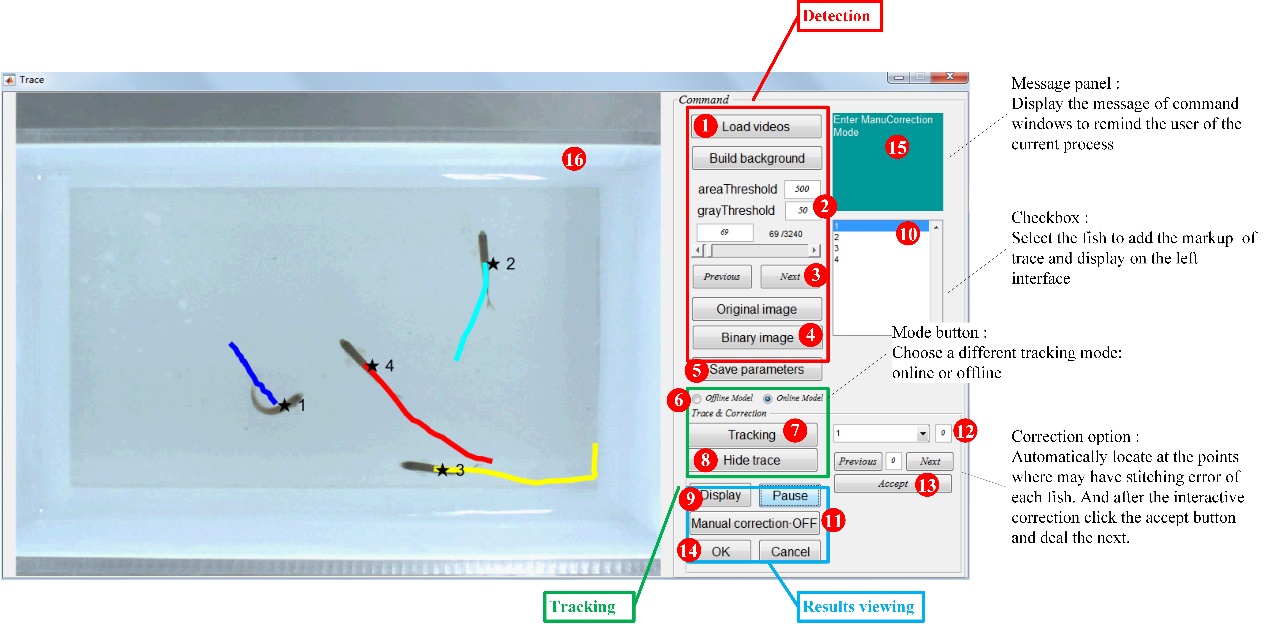


**Figure 3. Multi-target tracking platform GUI**

***Manual correction function:***

Manual correction • OFF button and Manual correction button, as well as the Link trace button can be click-converted by the mouse.

In the Manual correction button function, the left interface displays the trajectory of the targets in the past 20 frames and the next 3 frames based on the current frame. As the low confidence trajectory occurred in the crossing intervals, it is difficult to get the tracklets location by clicking mouse. Thus the start nodes and end nodes of the tracklets are marked with different symbols, then the mouse clicking can be easily carried out during the manual correction.

In the process of manual correction, the tracklets belonging to different identity with lower confidence are located in the lists. By selecting the different target IDs, the tracklets with lower confidence are located, and adjusting the orders of the frames by the Previous and the Next buttons to confirm whether the error occurs. If the tracklets with low confidence but with no matching error occurs, click the Accept button to automatically locate the next possible error point. If there is a matching error in the current frame, manual correction is performed. Use the right click of mouse to click on the fish body region or tracklets node points of tracklets before and after the crossing intervals, then the tracklets can be manual matched correctly. OK button to confirm the operation, Cancel button to cancel the current operation. Above all, manual error correction is implemented for all tracklets that may have the matching error.

Link trace function deals with the non-matched tracklets, in which the tracklets have not been matched due to the timing relationship after the algorithm is processed. Adjust the video frame before and after to select the matching target, then the process just as the manual error correction.

***Multi-target identifying***

To further verify the adaptability of the improved HOG feature, we also identified the drosophila (ISO4) and the black mouse (C57 B6) in the multi-target identifying experiment, in which drosophila wings were subtracted to be convenient for the sample images acquisition.

The target numbers for both drosophilae and black mouse were three. There were about 1500 frames in each fish video. The first 1000 frames in the video were used to train the classifier and the last 500 frames were used to test. Shown as **Table 1-2**, the identifying accuracy (IA) of the two groups of experiments was 100%, and the average classification accuracy (CA) were 91% and 73.63% respectively. In addition, there is no obvious grayscale gradient distribution in the mouse body after binarization since the uniform color of black mouse, so the HOG features is not obvious and the average classification accuracy is not high.

**Table1. Evaluation of drosophila identifying**

| **Category** | **CA** |
| --- | --- |
| C1 | 99.2% |
| C2 | 85.8% |
| C3 | 88% |

**Table2. Evaluation of mouse** identifying

| **Category** | **CA** |
| --- | --- |
| C1 | 80.23% |
| C2 | 64.6% |
| C3 | 76.10% |

In order to further verify the stability of the improved HOG feature, we identified 30 zebrafish (10 months old) in the growth cycle more than a month, the same 30 zebrafish were tested every week. In the experiment, thirty zebrafish were imaged separately. The detail data information of results is shown in **Table 3.**

**Table 3. Evaluation of zebrafish identifying in the growth cycle**

| **Duration** | **IA** | **maxCA** | **meanCA** |
| --- | --- | --- | --- |
| Week 1 | 100% | 97.74% | 80.88% |
| Week 2 | 80% | 100% | 66.46% |
| Week 3 | 63.33% | 95.59% | 61.98% |
| Week 4 | 63.33% | 97.74% | 64.98% |
| Week 5 | 63.33% | 100% | 63.70% |
| Week 6 | 60% | 91.04% | 58.63% |

***Multi-target tracking***

In the multi-target tracking experiment, we compared the proposed method and idTracker to track 11 videos with different numbers of zebrafish, the detail data information of results are shown in **Table 4.**

**Table 4. Evaluation of tracking results for different methods**

| **Dataset** | **Method** | **MissRate** | **ErrorRate** | **AccuracyRate** |
| --- | --- | --- | --- | --- |
| D1 | proposed | 2.04% | 0.30% | 99.71% |
|  | idTracker | 5.25% | 1.68% | 98.22% |
| D2 | proposed | 7.18% | 0.05% | 99.94% |
|  | idTracker | 7.45% | 3.61% | 96.09% |
| D3 | proposed | 4.76% | 0.56% | 99.37% |
|  | idTracker | 2.10% | 0.53% | 99.45% |
| D4 | proposed | 4.53% | 0.23% | 99.76% |
|  | idTracker | 6.55% | 1.69% | 98.10% |
| D5 | proposed | 7.32% | 0.47% | 99.49% |
|  | idTracker | 3.34% | 0.69% | 99.30% |
| D6 | proposed | 8.90% | 0.60% | 99.34% |
|  | idTracker | 4.74% | 1.19% | 98.75% |
| D7 | proposed | 3.59% | 3.35% | 96.53% |
|  | idTracker | 35.71% | 14.01% | 78.21% |
| D8 | proposed | 11.87% | 10.63% | 87.94% |
|  | idTracker | * | * | * |
| D9 | proposed | 0% | 0% | 100% |
|  | idTracker | * | * | * |
| D10 | proposed | 10.79% | 5.45% | 93.89% |
|  | idTracker | * | * | * |
| D11 | proposed | 0% | 0% | 100% |
|  | idTracker | 21.3% | 8.0% | 89.84% |

***Shoaling behavior of zebrafish***

In order to check whether the reserpine works before the shoaling behavior analysis, the locomotion behavior of zebrafish were measured by the total distance traveled, average velocity, turn angle and angular velocity in the novel tank. After 7 days with system water, the 9 treated fish and the 9 random fish in “WT” group were captured separately in the novel tank, the locomotion behavior parameters of them were measured by the total distance traveled, average velocity, turn angle and angular velocity to check whether the reserpine works. As shown in **Figure 4**, nine zebrafish treated with reserpine decreased significantly compared with the wild types in all parameters, which proved the zebrafish model of depression and anxiety-like behavior are effective before we observed whether the depression and hypoactivity introduced by reserpine manifested in fish shoal.

**
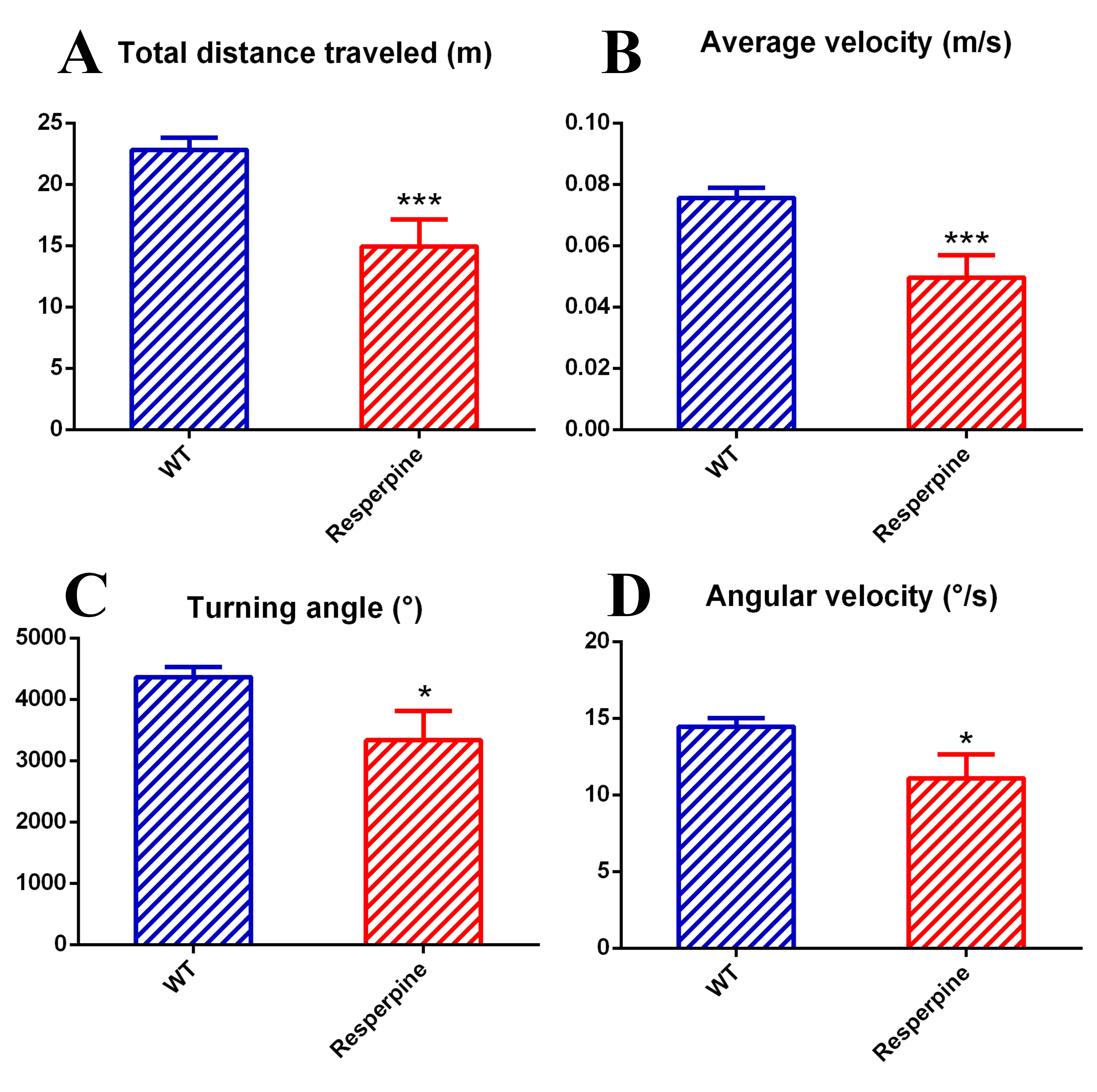
**

**Figure 4. The locomotion behavior parameters of the wild type (WT) and the depressive (Reserpine) model. (A) The total distance traveled of zebrafish treated with reserpine decreased significantly compared with the wild types. (B) The average velocity of zebrafish treated with reserpine decreased significantly compared with the wild types. (C) The angular velocity of zebrafish treated with reserpine decreased significantly compared with the wild types. (D) The turn angle of zebrafish treated with reserpine decreased significantly compared with the wild types.**
